# Correlation Between *PPARGC1A* Gene Rs8192678 G>A Polymorphism and Susceptibility To Type-2 Diabetes

**DOI:** 10.1515/biol-2019-0006

**Published:** 2019-05-21

**Authors:** Fei Du, Kang-Juan Yang, Lian-Shan Piao

**Affiliations:** 1 Department of Cell Biology and Medical Genetics, Yanbian University Medical College, Yanji, Jilin,133000, China; 2 Department of Endocrinology, Affiliated Hospital of Yanbian University, Yanji, Jilin,133000, China

**Keywords:** Type-2 diabetes mellitus, peroxisome proliferator-activated receptor γ coactivator 1α gene, single nucleotide polymorphisms, meta-analysis

## Abstract

**Objective:**

To systematically investigate the correlation between the G>A polymorphism of the peroxisome proliferator-activated receptor γ coactivator 1α (*PPARGC1A or PGC-1alpha*) gene rs8192678 locus and the susceptibility to type-2 diabetes mellitus (T2DM).

**Methods:**

The inclusion and exclusion criteria and retrieval strategies of original literatures were formulated. Then, subjects and free words “PPARGC1A”,”gene polymorphism”, and “T2DM” were retrieved from the PubMed, EMBASE, and Cochrane Library databases. Case-control studies on the G>A polymorphism of the *PPARGC1A* gene rs8192678 locus and susceptibility to T2DM were included for the meta-analysis.

**Results:**

The number of cases in the T2DM group and control group was 5,607 and 7,596, respectively. The meta-analysis revealed that the *PPARGC1A* gene rs8192678 locus G>A polymorphism is associated with susceptibility to T2DM. There are differences in each group of genetic models, of which three groups of genetic models are highly significant. In the allele model, OR=1.249, 95% CI: 1.099-1.419, and *P*=0.001. In the dominant inheritance model, OR=1.364, 95% CI: 1.152-1.614, and *P*=0.000. In the additive inheritance model, OR=0.828, 95% CI: 0.726-0.945, and *P*=0.005. And one group is significant, in the recessive inheritance model, OR=1.187, 95% CI: 1.021-1.381, and *P*=0.026.

**Conclusion:**

In Western Asian, South Asian, European and African populations, the A allele of the *PPARGC1A* gene rs8192678 locus may be one of the risk factors for T2DM.

Type-2 diabetes mellitus (T2DM) is a disorder of the metabolism of sugar, fat and protein caused by insulin resistance (IR) or the relative insufficiency of insulin secretion in islet beta cells, in which its occurrence and development are impacted by the dual factors of environment and heredity [1]. T2DM is a complex polygenic genetic disease, and the molecular and genetic mechanism of gene mutation, as well as the occurrence and development of T2DM or gene interaction, remain unclear. The expression products of the peroxisome proliferator-activated receptor gamma coactivator 1 alpha (*PPARGC1A or PGC-1alpha*) gene can regulate lipid secretion, fatty acid metabolism and insulin sensitivity [2]. Therefore, the *PPARGC1A* gene is a candidate gene for studying the metabolic syndrome and T2DM. The genetic variation at the 1444^th^ locus (rs8192678) in exon 8 of the *PPARGC1A* gene leads to G>A base substitution, causing the substitution of glycine by serine (Gly482Ser) in the amino acid sequence and structural change [3]. Initially, EK et al [4] used single strand conformational polymorphism analysis followed by nucleotide sequence to scan the *PPARGC1A* gene in 53 Danish T2DM patients. The study showed that the Gly482Ser polymorphism was associated with T2DM. Subsequently, Lacquemant et al [5] found that this locus was also significantly associated with T2DM in the British Caucasian population, but this association was not found in studies of the French Caucasian and Pima Indians. Moreover, there was no correlation between rs8192678 polymorphism and T2DM in East Asian Chinese population studies [6]. It was found that the association analysis of this locus with the T2DM case-control showed inconsistency in the population of many countries and regions. The inconsistency of this locus with T2DM suggests that statistical power may be reduced due to the small size of a single study, and random sample background or ethnic differences. It is difficult to determine the reasons for T2DM rs8192678 locus inconsistency using a single study. Meta-analysis by comprehensive analysis of all similar studies not only enhances statistical power and increases the credibility of gene polymorphisms in case-control association analysis, but also reduces random errors, false positives and false negatives. A meta-analysis of the *PPARGC1A* gene rs8192678 polymorphism and T2DM in recent years showed a significant association for this locus and T2DM in the Indian population (OR=1.19, 95% CI: 1.05-1.34, *P*=0.006). However, no significant associations were found among Caucasians or East Asians [7]. There are also meta-analysis results showing that the A allele of rs8192678 in the Chinese Han population of East Asia increases the risk of developing T2DM (OR=1.54, 95% CI: 1.34-1.81, *P*<0.001) [8]. Because the results of previous meta-analyses still have some differences, the heterogeneity between the studies is relatively large, especially in East Asia. Therefore, we believe that it is necessary to conduct a subgroup analysis by age and sample size of the method of applying meta-analysis to explore the cause of heterogeneity. The reason is to clarify the correlation between the *PPARGC1A* gene rs8192678 polymorphism and T2DM in different ethnicities, which will provide a basis of future biological functional research and clinical research of the *PPARGC1A* gene.

## 1 Materials and Methods

### 1.1 Literature retrieval strategy

Literature about the correlation between *PPARGC1A* gene polymorphism and T2DM was retrieved from the PubMed, EMBASE and Cochrane Library databases. Literature containing the subjects and free words “PPARGC1A”, “gene polymorphism”, and “T2DM” was retrieved from these databases. In addition, the cited references in relevant treatises and reviews were manually retrieved to collected case-control studies on the correlation between the G>A polymorphism of the *PPARGC1A* gene rs8192678 locus and susceptibility to T2DM. The retrieval time range was from the establishment of the databases to June 2018. By reading the titles and abstracts, and reading the full text when necessary, two investigators conducted an independent evaluation of the literature on the basis of the inclusion and exclusion criteria. When these two investigators disagreed with each other, all researchers in our team participated in the assessment of whether the literature should be included.

#### 1.1.1 All included literature must conform to the following criteria

(1) The study must be a case-control study, cohort study, or cross-sectional study on the association between *PPARGC1A* gene polymorphism and T2DM, and the case group should comprise of diabetic patients, who have combined diseases with no special restrictions. The control group should comprise of subjects with normal blood glucose levels and no family history of diabetes mellitus. (2) The literature must provide the genotype distribution frequency in the case group and control group, or the genotype distribution frequency could be calculated from the data provided by the literature. (3) If an article contains studies in two or more ethnic or regional populations, each study in one population was considered as one independent study. (4) The language in which the article was published was confined to English, and the sample size was unrestricted. For studies with incomplete data, the investigators did not intend to contact the authors.

#### 1.1.2 Exclusion criteria

Literature with incomplete data, literature in which the observed disease was not T2DM, literature in which the polymorphic loci did not meet the requirements, and literature with animal study subjects, reviews and meta-analysis studies were excluded.

### 1.2 Data extraction

After reading the full text of the literature, two investigators filled in a standard form with the following data in advance: author information, year of publication, ethnicity and country of the study subjects, the sample sizes of the case group and control group, the number of genotypes, and whether the genotype distribution in the control group was in accordance with the Hardy-Weinberg equilibrium (HWE). When these two investigators disagreed with each other, a third researcher would participate in determining the accuracy of the extracted data.

### 1.3 Quality evaluation of literature

The Newcastle Ottawa scale (NOS) was used to assess the quality of THE selected case-control studies, in which literature with a NOS score ≥5 was considered as high quality literature [9]. The Agency for Healthcare Research and Quality (AHRQ) score was used to evaluate the quality of cross-sectional studies, in which literature with an AHRQ score ≥8 was considered as high quality literature [10].

### 1.4 Data processing and statistical analysis

(1) Chi-square goodness of fit test was used to determine whether the distribution of genotypes in the control group in each study was in accordance with HWE. The odds ratio (OR) and 95% confidence interval (CI) were used to evaluate the correlation between *PPARGC1A* gene polymorphism and risk of T2DM in four different genetic inheritance models: allele model (A *vs*. G), dominant inheritance model (AA+GA *vs*. GG), recessive inheritance model (AA *vs*. GG+GA), and additive inheritance model (AA+GG *vs*. GA). (3) In order to determine whether there was significant heterogeneity among studies, the q statistic based on Chi-square test was used for qualitative analysis, while *I*^2^ statistic was used for quantitative analysis. If *I*^2^<50% and *P*>0.1, it was considered that there was no statistical heterogeneity among studies. Then, a fixed-effects model (M-H) was used for data consolidation, If *I*^2^≥50% and *P*<0.1, it was considered that there was statistical heterogeneity among studies. Then, a random-effects model (D-L) was used for data consolidation. (4) In order to explore for potential heterogeneity sources and robustness of the test results, a subgroup analysis was carried out based on ethnicity, sample size (number of case groups ≥300 or <300) and age (≥60 and <60), respectively. In order to explore the impact of a single study on the overall result, the step-by-step elimination method was used for the sensitivity analysis. That is, merely one study was eliminated at a time, and the effect size of the remaining studies was reconsolidated to observe the stability of results. If the OR value of the remaining studies was outside the range of the total effect size of 95% CI after one study was excluded, it was considered that the study results impacted the overall results. Finally, funnel plot and Begg’s method were used to detect for publication bias in the included literature. If the funnel plot was asymmetrical or *P*<0.05, publication bias was inferred.

## 2 Results

### 2.1 Literature retrieval results

**Figure 1:**
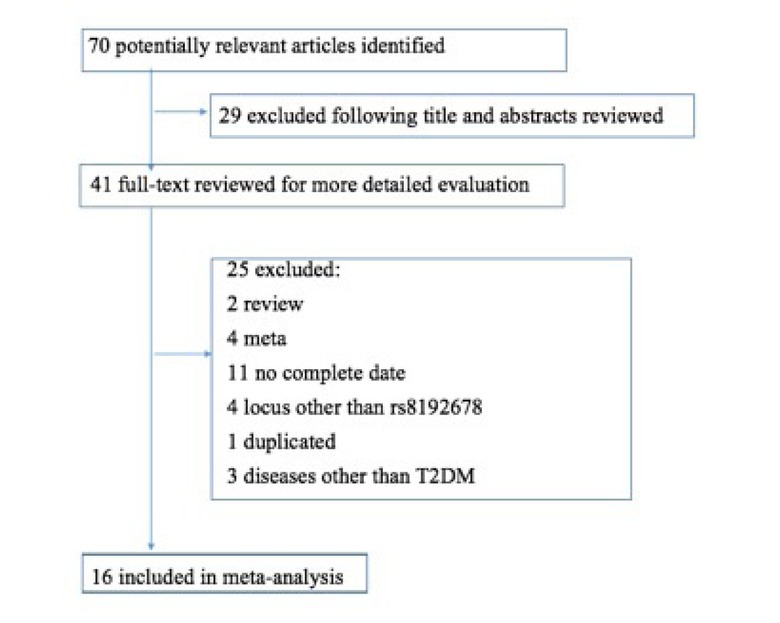
Retrieval flow chart of literatures for inclusion in the meta–analysis

According to the retrieval strategy, a total of 70 pieces of literature were primarily obtained. After reading the titles and abstracts, 29 irrelevant pieces of literature were excluded. Among the remaining 41 pieces of literature, after reading the full text, 25 literature pieces were excluded, which included two reviews, four meta analyses, 11 literature items with incomplete data, four literature items that did not study the gene polymorphic locus, one literature piece with suspected data duplication, and three literature items that studied diseases that were not T2DM. Finally, a total of 16 pieces of literature were included in the present meta-analysis [11, 12, 13, 14, 15, 16, 17, 18, 19, 20, 21, 22, 23, 24, 25]. The entire retrieval process is presented in Figure 1. These 16 pieces of literature included 12 case-control studies and five cross sectional studies, which comprised of 5,607 patients and 7,596 controls. The distribution of genotype in the control group for all the included studies was in accordance with the HWE. Four cross-sectional studies were evaluated as low and medium quality literature by AHRQ, and were excluded. The remaining literature was high quality (Table 1).

**Table 1:** Basic information on the association between *PPARGC1A* gene rs8192678 locus G>A polymorphism and type 2 diabetes mellitus

Author	Year	Ethnicity	Age		Cases	Controls	Genotypes (cases / controls)			HWE	Quality Score
			cases	controls	(n)	(n)	GG	GA	AA		
Pei et al	2017	East Asian	56.71±11.96	51.57±13.13	83	445	35	34	14	>0.05	8
							168	214	63		
Zhu et al.(1)	2017	East Asian	65.20±9.51	64.67±9.80	497	782	138	251	108	>0.05	5
							250	382	150		
Shokouhi et al.	2015	Wast	54.97±11.01	50.29±9.64	173	173	127	43	3	>0.05	7
		Asian									
							159	13	1		
Jemaa et al.	2015	African	43.60±12.20	49.10±9.50	487	402	166	231	90	>0.05	5
							176	170	56		
Weng et al.	2010	East Asian	60.30±8.40	54.50±10.80	276	1049	91	129	56	>0.05	7
							340	502	207		
Zhu et al.(2)	2009	East Asian	58.90±12.40	61.90±8.80	595	495	181	303	111	>0.05	6
							143	240	112		
Bhat et al. (group1)	2007	South	50.80±9.50	49.80±9.30	199	213	68	103	28	>0.05	5
		Asian									
							112	80	21		
Bhat et al. (group2)	2007	South	54.40±9.60	52.10±8.50	152	258	69	70	13	>0.05	5
		Asian									
							143	96	19		
Lu et al.	2007	East Asian	63.64±5.53	61.25±4.96	263	282	97	121	45	>0.05	8
							144	111	27		
Sun et al.	2006	East Asian	45.69±8.78	45.15±7.32	390	525	122	190	78	>0.05	7
							181	256	88		
Wang et al.	2005	East Asian	NS	NS	152	111	37	84	31	>0.05	6
							41	55	15		
Chen et al.	2004	East Asian	57.20±9.38	59.49±8.73	494	555	155	255	84	>0.05	7
							185	264	106		
EK et al.	2001	European	64.90±9.0	57.90±9.0	655	491	262	297	96	>0.05	7
							243	196	52		

### 2.2 Meta-analysis results

**Figure 2:**
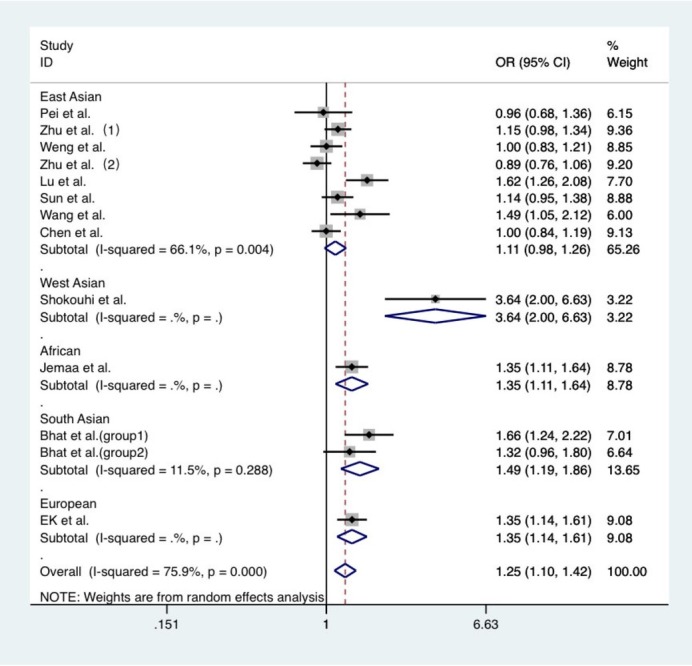
Forest map of allele models for the race subgroup analysis

A total of 12 case-control studies and one cross-sectional study, which involved 4,416 patients and 5,781 controls, were included in the meta-analysis. The results of meta-analysis revealed that the gene polymorphism rs8192678 locus was significantly correlated to T2DM in the general population. In the allele model, OR=1.249, 95% CI: 1.099-1.419, *P*=0.001, and *P*_heterogeneity_=0.000. In the dominant inheritance model, OR=1.364, 95% CI: 1.152-1.614, *P*=0.000, and *P*_heterogeneity_=0.000. In the recessive inheritance model, OR=1.187, 95% CI: 1.021-1.381, *P*=0.026, and *P*_heterogeneity_=0.077. In the additive inheritance model, OR=0.828, 95% CI: 0.726-0.945, *P*=0.005, and *P*_heterogeneity_=0.006. In addition, the aggregated data exhibited relatively robust heterogeneity, and a subgroup analysis was further applied to explore the sources of heterogeneity. Taking into account the differences in genetic backgrounds and environmental factors among different ethnicities, the same gene locus may have different effects on the same disease. Therefore, the investigators further explored the correlation in different ethnicities. The results revealed that in the allele model, gene polymorphism rs8192678 was associated with T2DM in the Western Asian population (OR=3.641, 95% CI: 2.000-6.628, *P*=0.000), South Asian population (OR=1.448, 95% CI: 1.187-1.865, *P*=0.001, *P*_heterogeneity_=0.288), European population (OR=1.354, 95% CI: 1.136-1.615, *P*=0.001), and African population (OR=1.351, 95% CI: 1.114-1.639, *P*=0.002), and the associations were all statistically significant. However, no significant correlation was detected in the East Asian population (OR=0.964, 95% CI: 0.685-1.358, *P*=0.106, *P*_heterogeneity_=0.004). In the dominant inheritance model, the correlation between gene polymorphism rs8192678 and risk of T2DM in Western Asian, South Asian, European and African populations was further confirmed. However, in the recessive inheritance model, no significant correlation between gene polymorphism rs8192678 and risk of T2DM was found in East Asian, West Asian, South Asian and African populations. In the additive inheritance model, no significant correlation between gene polymorphism rs8192678 and risk of T2DM was found in East Asian, European and African populations. The results of the meta-analysis on the correlation between rs8192678 and T2DM are presented in Table 2. A forest map of allele models for the ethnicity subgroups analysis is presented in Figure 2. The heterogeneity test revealed that there was greater heterogeneity among studies in the East Asian population, and the heterogeneity among studies in the South Asian population was smaller. However, a heterogeneity test could not be conducted in the remaining populations due to the small number of studies.

**Table 2:** Meta-analysis of rs8192678 gene polymorphisms and T2DM susceptibility

Genetic contrasts	Overall and subgroups	Study groups (n)	OR (95% CI)	Significance test		Heterogeneity		
				Z-value	P-value	Q-value	P-value	I2(%)
A versus G	All	13	1.249(1.099-1.419)	3.40	0.001	49.74	0.000	75.9
	East Asian	8	1.111(0.978-1.263)	1.61	0.106	20.63	0.004	66.1
	West Asian	1	3.641(2.000-6.628)	4.23	0.000	0.00	—	—
	African	1	1.351(1.114-1.639)	3.06	0.002	0.00	—	—
	South Asian	2	1.448(1.187-1.865)	3.44	0.001	1.13	0.288	11.5
	European	1	1.354(1.136-1.615)	3.38	0.001	0.00	—	—
(AA+GA) versus GG	All	13	1.364(1.152-1.614)	3.60	0.000	42.14	0.000	71.5
	East Asian	8	1.155(0.981-1.359)	1.74	0.083	15.17	0.034	53.9
	West Asian	1	4.114(2.164-7.818)	4.32	0.000	0.00	—	—
	African	1	1.506(1.147-1.976)	2.95	0.003	0.00	—	—
	South Asian	2	1.790(1.262-2.539)	3.27	0.001	1.52	0.217	34.4
	European	1	1.470(1.161-1.861)	3.20	0.001	0.00	—	—
(GG+GA) versus AA	All	13	1.187(1.021-1.381)	2.23	0.026	19.52	0.077	38.5
	East Asian	8	1.114(0.920-1.350)	1.10	0.269	14.18	0.048	50.6
	West Asian	1	3.035(0.313-29.470)	0.96	0.338	0.00	—	—
	African	1	1.401(0.974-2.014)	1.82	0.069	0.00	—	—
	South Asian	2	1.359(0.853-2.166)	1.29	0.197	0.25	0.619	0.0
	European	1	1.450(1.012-2.078)	2.02	0.043	0.00	—	—
(AA+GG) versus GA	All	13	0.828(0.726-0.945)	2.80	0.005	27.89	0.006	57.9
	East Asian	8	0.931(0.843-1.029)	1.40	0.162	5.56	0.592	0.0
	West Asian	1	0.246(0.127-0.476)	4.16	0.000	0.00	—	—
	African	1	0.812(0.622-1.060)	1.53	0.125	0.00	—	—
	South Asian	2	0.621(0.469-0.824)	3.30	0.001	0.55	0.459	0.0
	European	1	0.801(0.632-1.015)	1.83	0.067	0.00	—	—

### 2.3 Heterogeneity analysis

Since there was high heterogeneity among studies in the East Asian population, and in order to explore the source of heterogeneity in all genetic inheritance models, a subgroup analysis was carried out based on sample size (number of patients in the T2DM group was ≥300 or <300) and age (≥60 and <60). The results of the subgroup analysis revealed that after grouping, G>A polymorphism rs8192678 remained uncorrelated to susceptibility to T2DM (*P*>0.05). Except for the recessive inheritance model, when the sample size was ≥300, the heterogeneity among studies was significantly lower than before grouping. When the sample size was <300, the heterogeneity among studies was significantly higher than before grouping (Table 3). In the four models, when the age was ≥60, the heterogeneity among studies was significantly higher than before grouping. When age was <60, the heterogeneity among studies was significantly lower than before grouping (Table 4).

**Table 3:** rs8192678 gene polymorphisms and T2DM susceptibility in East Asian population based on sample size meta-analysis

Genetic contrasts	Overall and subgroups	Study groups (n)	OR (95% CI)	Significance test		Heterogeneity		
				Z-value	P-value	Q-value	P-value	I2(%)
A versus G	All	8	1.111(0.978-1.263)	1.61	0.106	20.63	0.004	66.1
	T2DM≥300	4	1.039(0.923-1.168)	0.63	0.528	5.60	0.133	46.6
	T2DM<300	4	1.230(0.935-1.618)	1.48	0.139	12.03	0.007	75.1
(AA+GA) versus GG	All	8	1.155(0.981-1.359)	1.74	0.083	15.17	0.034	53.9
	T2DM≥300	4	1.096(0.962-1.249)	1.38	0.168	2.42	0.490	0.0
	T2DM<300	4	1.263(0.855-1.866)	1.17	0.240	11.82	0.008	74.6
(GG+GA) versus AA	All	8	1.114(0.920-1.350)	1.10	0.269	14.18	0.048	50.6
	T2DM≥300	4	0.995(0.799-1.238)	0.05	0.961	6.18	0.103	51.4
	T2DM<300	4	1.354(0.983-1.864)	1.85	0.064	4.73	0.193	36.6
(AA+GG) versus GA	All	8	0.931(0.843-1.029)	1.40	0.162	5.56	0.592	0.0
	T2DM≥300	4	0.920(0.815-1.038)	1.36	0.174	0.85	0.838	0.0
	T2DM<300	4	0.954(0.757-1.202)	0.40	0.687	4.59	0.205	34.6

**Table 4:** rs8192678 gene polymorphisms and T2DM susceptibility in East Asian populations based on age meta-analysis

Genetic contrasts	Overall and subgroups	Study groups (n)	OR (95% CI)	Significance test		Heterogeneity		
				Z-value	P-value	Q-value	P-value	I2(%)
A versus G	All	8	1.111(0.978-1.263)	1.61	0.106	20.63	0.004	66.1
	Age≥60	3	1.211(0.950-1.545)	1.55	0.122	9.14	0.010	78.1
	Age<60	4	0.996(0.894-1.110)	0.07	0.942	3.58	0.311	16.1
	NS	1	1.489(1.048-2.118)	2.22	0.027	0.00	—	—
(AA+GA) versus GG	All	8	1.155(0.981-1.359)	1.74	0.083	15.17	0.034	53.9
	Age≥60	3	1.269(0.926-1.739)	1.48	0.139	7.16	0.028	72.1
	Age<60	4	1.028(0.888-1.190)	0.37	0.711	2.23	0.526	0.0
	NS	1	1.820(1.067-3.107)	2.20	0.028	0.00	—	—
(GG+GA) versus AA	All	8	1.114(0.920-1.350)	1.10	0.269	14.18	0.048	50.6
	Age≥60	3	1.256(0.928-1.700)	1.48	0.139	4.27	0.118	53.2
	Age<60	4	0.964(0.763-1.219)	0.30	0.761	4.99	0.172	39.9
	NS	1	1.640(0.837-3.211)	1.44	0.149	0.00	—	—
(AA+GG) versus GA	All	8	0.931(0.843-1.029)	1.40	0.162	5.56	0.592	0.0
	Age≥60	3	0.931(0.796-1.089)	0.90	0.370	2.07	0.355	3.5
	Age<60	4	0.943(0.821-1.083)	0.83	0.407	3.06	0.382	2.0
	NS	1	0.795(0.487-1.299)	0.92	0.360	0.00	—	—

### 2.4 Results of the sensitivity analysis and publication bias

**Figure 3:**
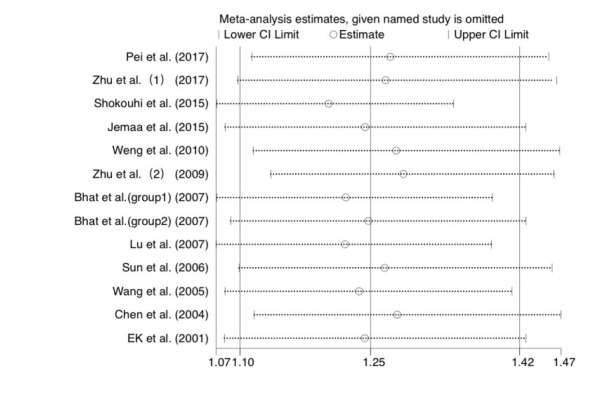
Sensitivity analysis

**Figure 4:**
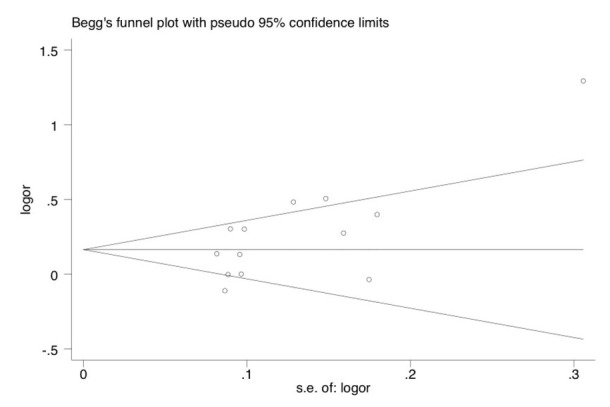
Begg’s funnel plot

As shown in Figure 3, after the 13 studies were excluded one by one, the meta-analysis results for the remaining 12 studies did not significantly change. This indicates that the results of the meta-analysis were stable. As shown in Figure 4, no publication bias was found in the present study (Begg’s test: *P*=0.059).

## 3 Discussion

*PPARGC1A* is a highly conservative transcriptional co-activator, which is abundant in metabolic active tissues, such as the liver, fat, pancreas and muscles. It plays a role in regulating energy metabolism in the whole

body. The single nucleotide variation of the *PPARGC1A* gene rs8192678 is correlated to susceptibility to T2DM, the relative risk of obesity and insulin resistance, and lower β-cell function index [6]. Research on the correlation between *PPARGC1A* gene G>A polymorphism rs8192678 and susceptibility to T2DM has gradually become a focus of attention. However, there were inconsistencies in these studies. The results of the present meta-analysis revealed that in non-grouped populations, *P*^heterogeneity^=0.000. In the allele model, OR=1.249, 95% CI: 1.099-1.419, *P*= 0.001, and *P*_heterogeneity_=0.000. In the dominant inheritance model, OR=1.364, 95% CI: 1.152-1.614, *P*=0.000, and *P*_heterogeneity_=0.000. In the recessive inheritance model, OR=1.187, 95% CI: 1.021-1.381, *P*=0.026, and *P*_heterogeneity_=0.077.

In the additive inheritance model, OR=0.828, 95% CI: 0.726-0.945, *P*=0.005, and *P*_heterogeneity_=0.006. These results reveal that the A allele of the *PPARGC1A* gene rs8192678 locus is susceptible to T2DM. Since the heterogeneity among studies was high, subgroup analysis was carried out based on the ethnicity of the population. The results suggest that the A allele was associated with the incidence of T2DM in Western Asian, South Asian, European and African populations (*P*<0.05), but was not associated with T2DM in East Asian population (*P*>0.05). The meta-analysis of Yang et al showed a significant association for this locus and T2DM in the Indian population (OR=1.19, 95% CI: 1.05-1.34, *P*=0.006) [7]. However, no significant findings were found in the East Asian population. The association is consistent. But meta-analysis with Chen et al showed that the A allele of rs8192678 in Chinese Han population in East Asia increased the risk of T2DM (OR=1.54, 95% CI: 1.34-1.81, *P*<0.001) [8]. Since there was heterogeneity among studies in the East Asian population, patients were grouped according to the number of cases and age of the T2DM group, but no positive correlation was found between the A allele and the incidence of T2DM. However, when the number of samples was ≥300 and age was <60, the heterogeneity was significantly reduced. Studies with less than 300 cases probably do not have enough statistical power to obtain OR~1.5 with allelic frequencies between 0.20 and 0.40 in case-control design (see in Quanto software). It is possible that part of the heterogeneity found in this group is because of lack of statistical power. Ling et al [26] concluded that insulin stimulates and aging reduces skeletal muscle expression of *PPARGC1A* and *PPARGC1B*, and suggested that they have different regulatory functions on glucose and fat oxidation in muscle cells. The authors suggested that this could provide an explanation by which an environmental trigger (age) modifies genetic susceptibility to T2DM. Heterogeneity at ages greater than 60 years may be related to environmental factors.

An interesting aspect is the A allele frequency in the East Asian populations is 44%, more than 15% in relation to South Asian, 8% of the European population and more than 39% of the African population. This may be due to the fact that this variant does not influence T2DM due to adaptive issues (See www.internationalgenome.org/1000-genomes-browsers/) Another possibility is about the linkage disequilibrium (LD). In LD-based indirect correlation analysis, if a disease-causing locus and genetic markers (polymorphic alleles) have strong LD, then it can be compared to normal individuals by comparing genetic markers. Differences ultimately lead to the relative risk of disease-causing loci in the disease. If LD between the SNP and the causal loci is weaker in East Asian than in South Asian or Europeans, it may lead to a weaker association which may not be detected [27]. If the size effect is very low in East Asian population, it could only be detected by increasing the statistical power with a larger sample size.

In the present study, strict inclusion and exclusion criteria were designed, a stratified analysis was conducted to deal with the confounding bias in the study, and the credibility of results of the analysis was satisfactory. Nevertheless, the present study had certain limitations: (1) the T2DM diagnosis was drawn in most of the included studies, according to the Diagnostic Criteria of Diabetes published by the World Health Organization (WHO), but this was drawn according to the Diagnostic Criteria of Diabetes published by the American Dental Association (ADA) in some studies, which may result in some differences among patients who were included in these studies; (2) merely the *PPARGC1A* gene polymorphism of one locus was analyzed, and although the results of the analysis revealed a statistically significant correlation, the number of cases in each study was small, and the potential interactions between gene-gene and gene-environment were not included in the present meta-analysis, showing that the results of the analysis could only be used for reference.

In summary, *PPARGC1A* gene G>A polymorphism rs8192678 may increase the risk of T2DM in Western Asian, South Asian, European, and African populations, while this was not correlated to susceptibility to T2DM in the East Asian population. Taking into account that the occurrence of T2DM is the result of the combined action of genetic and environmental factors, and some limitations in the present study, the exact conclusions need to be further verified through large sample case-controlled or prospective clinical studies.
